# Microplastic Abundance and Sources in Surface Water Samples of the Vaal River, South Africa

**DOI:** 10.1007/s00128-023-03845-y

**Published:** 2024-01-05

**Authors:** Dalia Saad, Gibbon Ramaremisa, Michelle Ndlovu, Patricia Chauke, Josiane Nikiema, Luke Chimuka

**Affiliations:** 1 School of Chemistry, Molecular Sciences Institute, University of the Witwatersrand, Johannesburg, South Africa; 2 Department of Chemistry, University of Pretoria, Pretoria, South Africa; 3 International Water Management Institute, Accra, Ghana

**Keywords:** Microplastics, Freshwater, Vaal River, South Africa

## Abstract

Microplastics (MPs) have emerged as a global environmental concern due to their persistent nature. In South Africa, microplastic research has primarily focused on marine systems. However, recent years have seen a shift in focus to studying MPs in South African freshwaters. In this study, MPs with a minimum size of 0.055 mm in surface water of the Vaal River, South Africa, were reported. MPs were 100% prevalent, with a mean numerical abundance of 0.68 ± 0.64 particles/m^3^. Small-sized MPs of < 1 mm accounted for the largest proportion. MPs were chemically identified as high-density polyethylene, low-density polyethylene, and polypropylene according to their Raman spectra. The prevalence of fragments (41.6%) and fibers (38.5%) over pellets (8.1%) indicates that microplastics are from secondary sources. The prevalence of polyethylene and polypropylene is consistent with microplastics being from secondary sources. These polymers are commonly used in single-use plastics, packing bags, textiles, and containers. These characteristics are of great concern due to their implications on the bioavailability and toxicological impacts of MPs. Consequently, these properties may pose more hazards to aquatic biota inhabiting the Vaal River.

Microplastics (MPs) result from the breakdown of larger pieces of plastics as well as manufactured MPs (Park and Park 2021). Microplastics enter the aquatic environment from different sources, including effluents from wastewater treatment plants (WWTPs), sewage sludge, shipping activities, atmospheric fallout, direct disposal from the public, beach littering, and runoff from agricultural, recreational, industrial, and urban areas (Nikiema et al. 2020a, 2000b ; Bellasi et al. 2020). They are divided into two categories, namely primary (manufactured plastic products produced at a microscopic size) and secondary (small plastic particles that are formed by the breakdown of larger plastic items) (Sun et al. 2019; Onoja et al. 2022).

Environmental contamination by MPs is of great concern. In addition to their possible health risks, the potential hazard associated with MPs includes exposure to their constituent additives such as plasticizers, flame retardants, pigments, and stabilizers (Li et al. 2023). Further, MPs have the potential to act as micro-vectors for toxic elements and organic contaminants, adsorbing and desorbing them depending on the prevailing environmental conditions (Onoja et al. 2022). Plastics are also known to be good adsorbents of some trace elements e.g. mercury, lead, and cadmium among others (Liu et al. 2023). This capability varies depending on the functional groups pendent on the backbone structure of the plastic (Saad 2022a). MPs are reported to interact with aquatic organisms including plants. Alterations in tissue morphologies, gene expression, enzyme activity, swimming patterns, and kidney functions are some of the toxicological effects reported in several aquatic biota (Saad 2023).

MP research has primarily focused on marine systems, however, the past few years have seen a shift in focus to studying MPs in South African freshwaters (Dahms et al. 2020; Saad et al. 2022b; Weideman et al. 2019). South Africa has a well-established plastics manufacturing sector, however, recycling is insufficient. It is one of the top twenty countries that produce large masses of plastic waste, which ends up in aquatic environments. Yet, MP pollution in the freshwater of South Africa has not been thoroughly investigated (Saad et al. 2022b). This study aimed to investigate the presence, abundance, and sources of MPs in one of the most essential freshwater systems in South Africa, the Vaal River. The main objective was to report the concentration levels of MPs in surface water, using a volume-reduced sampling method, for different areas of the Vaal River system.

## Materials and Methods

Sampling was conducted in June 2021 in Vereeniging, a city located in the south of Gauteng province, bordering the Free province. Samples were collected from the middle part of the Vaal River over 50 km, between the Lethabo weir and the Vaal River barrage (Fig S1). This region of the Vaal is known for different anthropogenic activities including industrial, agricultural, and recreational activities. Eskom–Lethabo power station and Rand Water Lethabo pump station are also located in this area. This part of the river receives water flowing from five tributaries and discharges from WWTPs. Sampling was conducted using a plankton net with 0.25 m, 50 cm, and 0.055 mm diameter, length, and mesh size, respectively. Twenty surface water samples were collected, and the sampled material was concentrated inside a removable net bucket with a sieve gauze-covered side window and stop-cock, through which it was transferred into glass jars. GPS and flowmeter readings at the beginning and the end of each tow were recorded and the volume filtered through the net was calculated using a flowmeter (General Oceanics model 2030R) attached to the net (Ramaremisa et al. 2022). Samples were stored in glass jars, transferred to the laboratory, and preserved at 4 °C.

Samples were digested by wet peroxide oxidation process (WPO) using Fenton’s reagent (0.07 M FeSO_4_·7H_2_O catalyst and 30% (v/v) H_2_O_2_). 60 mL of Fenton’s reagent was added to each sample in a 1:1 volume ratio. The catalyst solution was added first and the H_2_O_2_ solution was slowly added, until no further reaction. The samples were subsequently swirled to allow the solution to soak into the sample. Samples were left for 24–48 h at room temperature to achieve complete digestion before separation (Ramaremisa et al. 2022).

Upon digestion, MPs were separated based on their density using both NaCl and NaI, in a two-step density separation protocol (Ramaremisa et al. 2022). Firstly, 45 mL of saturated NaCl (1.2 g/mL) was mixed with each sample, left for 24 h, and filtered. Subsequently, 45 mL of NaI (1.8 g/mL) was added and left for another 24 h before filtration. The two-step protocol ensures high extraction of high-density MPs while avoiding excessive use of NaI. Samples were then vacuum filtered through GF/A filter papers (Whatman® : 47 mm diameter, pore size: 1.6 μm), air-dried in a fume hood, and stored in covered glass Petri dishes until analysis.

Following the extraction, the physical properties of potential MP particles were identified using a Nikon stereomicroscope (Nikon MET SMZ745T, Japan) at 50x magnification. Particles suspected to be MPs were photographed with an imaging source camera (TIS) USB 3.0 and processed with NIS Elements-D imaging software Version 5.30 (Nikon Cooperation, Japan). In addition to identifying their shape and colour, the software was also used to count particles as well as measure particle size. MPs were grouped into six size classes: <0.5 mm, 0.5–1 mm, 1–2 mm, 2–3 mm, 3–4 mm, and 4–5 mm. MPs were identified based on the criteria proposed in the literature, namely, a maximum size of 5 mm, absence of biological structures, and homogeneous colours (Ramaremisa et al. 2022).

The polymer type of MPs in this study was identified using Raman spectroscopy. Frequently observed MP particles were analysed by a Horiba LabRAM HR Raman spectrometer with an Olympus BX41 microscope attachment, using a 514.5 nm (green) line of a Lexel Model 95-SHG argon ion laser. LabSpec v5 software is used for acquisition and analysis of spectra, the spectra ranged from 0 to 3500 cm^1^ and the wavelength of the incident laser was set to 1–2 μm. The chemical composition of polymers was identified by comparison with reference spectra in the SLOPP Library of Microplastics and polymer databases of KnowItAll software (Bio-Rad Laboratories, Inc.).

Extreme caution was taken during sample collection, sample processing, and analysis to prevent cross-contamination. Except for the plankton net, no plastic apparatus or containers were used during sampling and sample preparation, and experimental equipment and containers were washed with filtered Milli-Q® Type 1 Ultrapure water and rinsed with filtered ethanol. All laboratory experiments were conducted in a laminar flow cabin in a lab dedicated to MPs, and cotton lab coats were worn during all stages to prevent contamination risk from clothing fibers. To mitigate against the potential contamination of samples by MPs in chemicals, all solutions were filtered through a Whatman® GF/F glass microfiber filter (Cytiva Danaher Group, Buckinghamshire, United Kingdom) before use. In addition, blank experiments were conducted with filtered Milli-Q® Type 1 Ultrapure water to count for possible procedural contamination. All blanks were processed along with the samples. No MPs were detected in the blanks.

## Results and Discussion

**Fig. 1:**
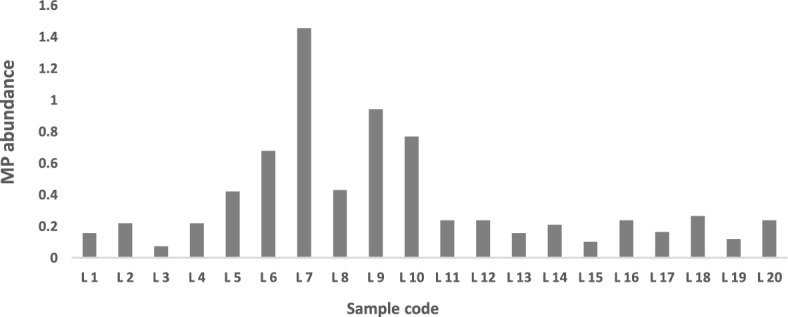
MPs abundance per sample

All samples showed the presence of MPs with a mean abundance of 0.68 ± 0.64 particles/m^3^. Abundance per sample location is given in Fig. 1.

Higher abundance was detected at locations L5, L6, L7, L8, L9 and L10 with over 0.4 particles/m^3^ each. It is noted that MP pollution is attributed to several factors, most significantly, the proximity of the water body to highly populated urban areas, industrial hubs, (WWTPs), and other anthropogenic activities such as tourism, fishing, and agriculture (Saad et al. 2022c). These sampling spots are located between Vereeniging and Vanderbijlpark (Fig S1), where potential sources of MPs exist. Potential sources in the area include industries (Arcelor Mittal Vaal works and Vaal Rubberising Centre, which are located near L6); Water and wastewater treatment works (L6 is 1.5 km from Rand water Vereeniging pump station); urban settlements (L 8 and L 9 are near the North–West University Vaal Triangle campus and Vaal University of Technology in Vanderbijlpark); recreational activities (L6 is less than 1 Km from Riviera aquatics club, L9 is near Emerald Resort animal world, and the Aquadome and Eligwa boat club are located close to L10); informal settlements (L 7 is near to informal human settlements under the R59 road). Informal settlements are profoundly marked by environmental degradation and pollution due to illegal dumping, poor waste management, and logistical challenges (Verster and Bouwman 2020; Grangxabe et al. 2023; Kekana et al. 2023). This explains the high abundance of MPs in L7 .

In addition, L9 and L10 are approximately 1.4 km and 1.176 km away from the confluence of the Vaal River and Taaibosspruit River. The increase of MPs in main rivers at confluences of tributaries is well documented (Dahms et al. 2020). Further downstream a sharp increase is observed at L16, which is about 1 km away from the confluence of the Vaal River with Leeuspruit River. However similar observations were not made at the confluence of the Vaal River and Rietspruit, this could be due to the Vaal-Rietspruit River confluence being stretched out into a Lake (Loch Vaal) as shown in Fig S2. Subsequently, MPs may be more abundant further away from the area sampled.

**Fig. 2:**
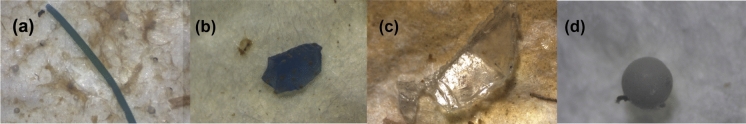
Microscope images of different shapes: **a** fiber, **b** fragment, **c** film and **d** pellet

Morphological characteristics (shape, color, and size) of MP particles were analysed. Microscope images of the most frequently detected shapes are shown in Fig. 2.

**Fig. 3:**
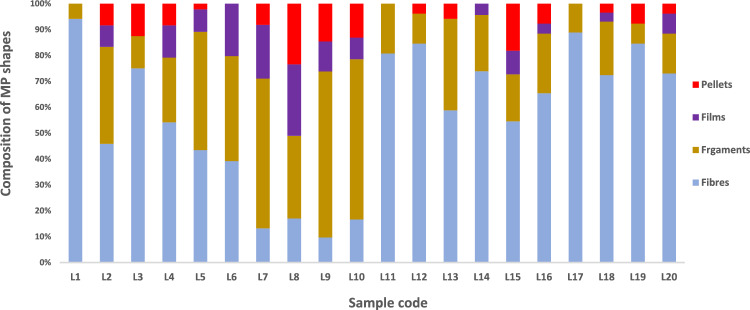
Distribution of shapes

Fragments (41.6%) and fibers (38.5%) dominated other shapes. Films and pellets were less-existent, representing only 11.8% and 8.1%, respectively (Fig. 3). The high prevalence of fragments and fibers over pellets indicates that MPs are from secondary sources Ramaremisa et al. 2022).

Domestic discharge from households around the Vaal River may contribute immensely to the prevalence of fibrous MPs, mainly, from machine washing (Lahiri et al. 2023). Sun et al. (2019) investigated the pervasiveness of fibers in the treatment stages and reported the persistence of fibers during wastewater treatment processes. Notably, fibers and fragments are frequently reported as the most abundant shapes of MPs in several freshwater bodies, examples include the Karnafully River in Bangladesh, the Chengdu River in China, and several freshwater lakes and Rivers in Russia (Chen et al. 2022; Frank et al. 2022; Hossain et al. 2022). Weideman et al. (2019) reported a high abundance of fibers (98%) in bulk water samples.

**Fig. 4:**
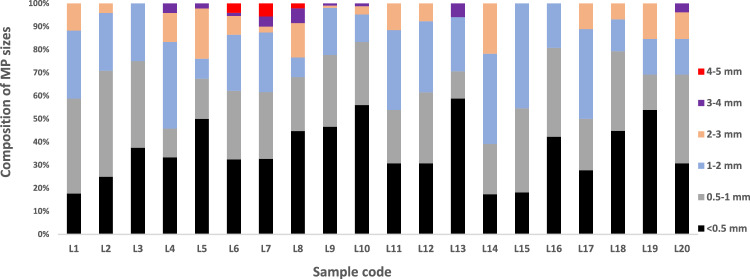
Distribution of sizes

In terms of size, it is observed that smaller sizes less than 0.5 mm were the most abundant, accounting for 38.8% of the total particles, followed by 28.3% of MPs in the range 0.5–1 mm, and 22.1% in the range 1–2 mm. While larger particles (2–5 mm) are less abundant and represent 10.7% of the total particles detected in all samples. Figure 4 shows the size distribution of MPs in the surface water samples. In riverine systems, MPs are susceptible to environmental weathering such as phyto, thermal, microbial, and chemical degradation. These degradation processes result in a continuous decrease in MP size over time (Bellasi et al. 2020; Onoja et al. 2022; Ramaremisa et al. 2022), which explains the dominance of small-sized MPs in this study. The dominance of small-sized MPs has been reported widely (Govender et al. 2020; Ramaremis et al. 2022; Saad et al. 2022a, 2022b; Saad and Alamin 2024).

**Fig. 5:**
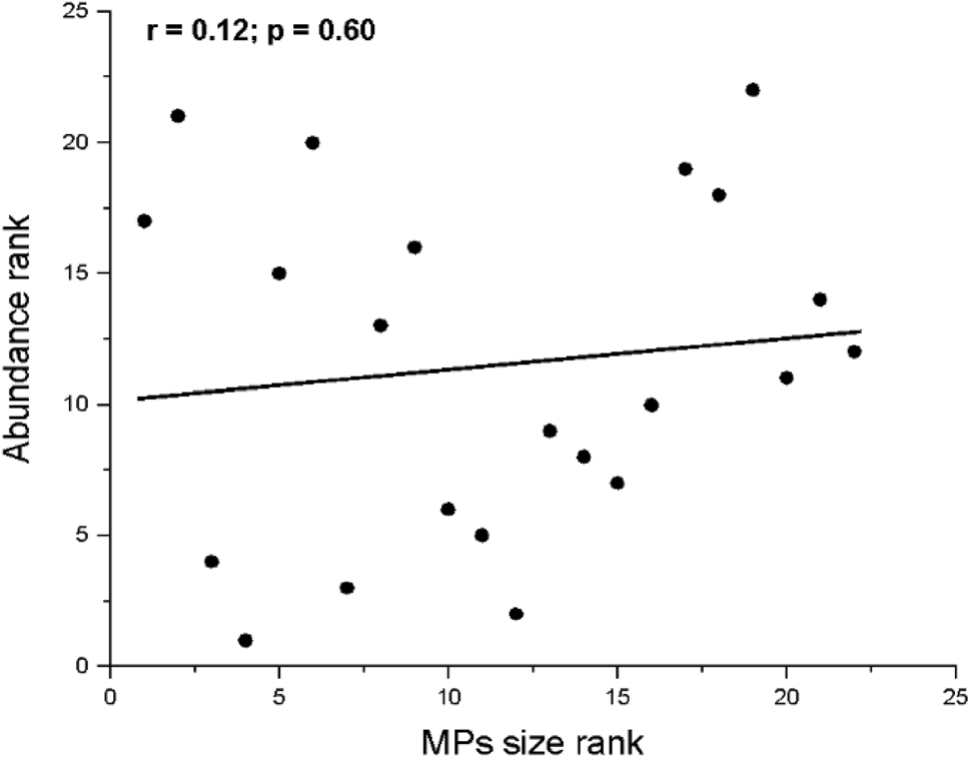
Spearman correlation plot

The relationship between MP abundance and their size was examined by the Spearman correlation test (Fig. 5). A p-value of 0.6 indicates an insignificant correlation. This could be explained by the fact that MP size is affected by the environmental degradation processes mentioned above and not directly linked to the abundance.

Coloured MPs were found to be more abundant (82.4%) compared to transparent MPs (17.6%) as shown in Fig S3. The dominance of coloured MPs has been reported in several freshwater studies around the world (Zhao et al. 2015). This is obvious as plastics are commonly coloured during manufacturing to enhance their appearance for different applications.

Post-visual identification, the chemical composition of MPs was further examined by Raman spectroscopy. The most frequent MP particles were identified as high-density polyethylene (HDPE), low-density polyethylene (LDPE), and polypropylene (PP), according to their Raman spectra (Fig S4). Differences between sample spectra and library spectra may reflect one or a combination of the following factors, the presence of chemical additives in the MPs, partial polymer degradation, and the presence of residues of biofilms that had colonised the plastic surfaces over time (Frère et al. 2016; Käppler et al. 2016). PE and PP were frequently reported in MP monitoring studies in surface water, which could be attributed to their extensive use in manufacturing different products. Additionally, the low density of these polymers makes them buoyant, thus abundant in the surface water (Govender et al. 2020). The polymer types, therefore, influence their distribution and transport in aquatic environments based on their density. While high-density polymers are known to be bioavailable for benthic organisms, low-density polymers such as PE and PP are consumed by pelagic organisms such as plankton. However, due to biofouling, low-density polymers can sink and become bioavailable for benthic biota. Benthic organisms can also consume low-density MPs via trophic transfer (Lagarde et al. 2016; Long et al. 2015).

Several adverse effects, including physical damage and changes, oxidative stress, and immune and gene destructions, are observed in organisms that consumed PE MPs (Hamed et al. 2022; Rochman et al. 2014).

The abundance of MPs in this study is moderate in comparison with other freshwater studies. Worldwide freshwater studies were selected to compare with the current study (Table 1).

**Table 1:** Abundance of MPs in different freshwater bodies

Freshwater system	Sampling apparatus	Mean MP abundance	Refs.
Lijiang river (China)	0.3 mm plankton net	0.15 ± 0.15 particles/m^3^	Zhang et al. 2021
Beijiang River (China) Lake Bolsena (Itally)	0.112 mm plankton net 0.3 mm manta trawl	0.56 ± 0.45 particles/m^3^ 0.82–4.42 particles/m^3^	Tan et al. 2019 Fischer et al. 2016
Ofanto River (Italy)	0.3 mm plankton net	0.9 particles/m^3^	Campanale et al. 2020
The Pearl River (China)	0.3 mm manta trawl	2.4 ± 0.70 particles/m^3^	Lam et al. 2020
Lake Chiusi (Italy)	0.3 mm manta trawl	2.68–3.36 particles/m^3^	Fischer et al. 2016
The Vaal River (South Africa)	0.055 mm plankton net	0.68 ± 0.64 particles/m^3^	This study

The values show that the level of MP abundance in the Vaal River was higher than those reported in two Chinese freshwater systems. Higher levels of MP contamination were reported in three Italian freshwater bodies and another two Chinese Rivers. However, the comparison of MP abundance among different studies must be performed with some caution, due to significant methodological differences in sampling, sample preparation, and visual identification. The Vaal River is exposed to discharges from industrial, mining, power generation, commercial agriculture, nature conservation, as well as urban and rural human settlements (Saad et al. 2022b). Fishing activities, domestic sewage, poor plastic waste management, population densities, and different anthropogenic activities are considered to be the major contributors to MPs abundance most of these cases.

## Conclusions

Surface water of the Vaal River were studied to document the presence, abundance, and the sources of MPs in the Vaal River. The abundance of MPs is comparable to the findings of some freshwater studies from other parts of the world. Fragments and fibers represented over 80% of the detected MPs, the majority of which were coloured particles of sizes below 0.5 mm. Polymer types were identified as HDPE, LDPE, and PP. Different anthropogenic activities were identified as potential sources of MPs in the Vaal River including, industries, tourism, fishing, and agriculture.

This report on the status of MP abundance in surface water of the Vaal River provides a reference for further monitoring studies, which may assist in extending our knowledge of MP pollution in South African freshwater systems.

## Data Availability

Data are available upon request.
